# PLD1 is a key player in cancer stemness and chemoresistance: Therapeutic targeting of cross-talk between the PI3K/Akt and Wnt/β-catenin pathways

**DOI:** 10.1038/s12276-024-01260-9

**Published:** 2024-07-01

**Authors:** Seong Hun Lim, Hyesung Lee, Hyun Ji Lee, Kuglae Kim, Junjeong Choi, Jung Min Han, Do Sik Min

**Affiliations:** 1 Department of Pharmacy, Yonsei University, Incheon, 21983 Republic of Korea; 2 POSTECH Biotech Center, Pohang University of Science and Technology, Pohang, 37673 Republic of Korea; 3 Yonsei Institute of Pharmaceutical Sciences, College of Pharmacy, Yonsei University, Incheon, 21983 Republic of Korea

**Keywords:** Cancer, Cell biology

## Abstract

The development of chemoresistance is a major challenge in the treatment of several types of cancers in clinical settings. Stemness and chemoresistance are the chief causes of poor clinical outcomes. In this context, we hypothesized that understanding the signaling pathways responsible for chemoresistance in cancers is crucial for the development of novel targeted therapies to overcome drug resistance. Among the aberrantly activated pathways, the PI3K-Akt/Wnt/β-catenin signaling pathway is clinically implicated in malignancies such as colorectal cancer (CRC) and glioblastoma multiforme (GBM). Aberrant dysregulation of phospholipase D (PLD) has been implicated in several malignancies, and oncogenic activation of this pathway facilitates tumor proliferation, stemness, and chemoresistance. Crosstalk involving the PLD and Wnt/β-catenin pathways promotes the progression of CRC and GBM and reduces the sensitivity of cancer cells to standard therapies. Notably, both pathways are tightly regulated and connected at multiple levels by upstream and downstream effectors. Thus, gaining deeper insights into the interactions between these pathways would help researchers discover unique therapeutic targets for the management of drug-resistant cancers. Here, we review the molecular mechanisms by which PLD signaling stimulates stemness and chemoresistance in CRC and GBM. Thus, the current review aims to address the importance of PLD as a central player coordinating cross-talk between the PI3K/Akt and Wnt/β-catenin pathways and proposes the possibility of targeting these pathways to improve cancer therapy and overcome drug resistance.

## PLD Signaling in Cancer: Unveiling Pathways to Overcome Chemoresistance

Cancer coming back after it seemed to have gone away is a big problem in treating cancers like colorectal cancer and a brain cancer called glioblastoma multiforme. This research looks at the part played by cancer stem cells (CSCs - cells within a tumor that can self-renew and cause the cancer to grow and come back) in cancer coming back and not responding to treatment. The scientists found that a pathway in the cells, called the Wnt/β-catenin signaling pathway, is important for keeping CSCs going. They also found that an enzyme (a type of protein that speeds up reactions in the body) called phospholipase D1 (PLD1) helps control this pathway. By stopping PLD1, they could lower the ability of CSCs to keep renewing themselves and make them more responsive to chemotherapy. This means that focusing on PLD1 could be a new way to treat cancers that don’t respond to existing treatments.

This summary was initially drafted using artificial intelligence, then revised and fact-checked by the author.

## Introduction

One of the current challenges in comprehensive cancer treatment is recurrence, which occurs after a period of remission, thereby contributing to treatment failure and disease progression. Colorectal cancer (CRC) continues to be one of the main causes of cancer-related deaths because patients exhibit disease progression and a poor prognosis related to resistance to current therapies. Glioblastoma multiforme (GBM) is a devastating primary glial brain tumor; its overall prognosis remains dismal, and there is an unmet need for effective therapeutic strategies. Within the tumor bulk, there is a small population of neoplastic units, termed cancer stem cells (CSCs), and these cells play a vital role in CRC or GBM tumorigenesis, progression, maintenance, invasion and recurrence^1–4^. Although the oncogenic roles of CSCs have not yet been completely elucidated, these slowly dividing cells are different from rapidly dividing tumor cells and are difficult to target using conventional therapies without harming adjacent nonneoplastic tissues^5^. Therefore, it is important to understand the mechanisms through which CSCs cause recurrence from several perspectives. The CSC microenvironment stimulates signaling pathways such as the Wnt, Notch, and Hedgehog pathways to facilitate metastasis, invasion, and anoikis evasion^6–8^. Among the various signaling pathways involved in CSC-driven chemoresistance, aberrant activation of Wnt signaling appears to be essential for the maintenance of CSC populations, consequently altering patient clinical outcomes. Remarkably, 90% of CRC patients exhibit aberrant activation of the canonical Wnt/β-catenin signaling pathway^9,10^. Dysregulation of the Wnt/β-catenin pathway plays a fundamental role in the genesis and progression of several types of cancer, including CRC and GBM^10–12^. The Wnt pathway is strictly regulated under physiological conditions and modulates fetal development and homeostasis in adult tissues^10^. Glioblastoma stem cells (GSCs) play important roles in tumor formation by activating multiple signaling pathways. The Wnt signaling pathway is an important pathway that aids cellular differentiation to promote tumor formation in the brain. Recently, the Wnt/β-catenin signaling pathway has emerged as a significant determinant of GBM initiation and progression^13^. Wnt signaling is also crucial for regulating GSCs, which makes it an attractive target for GBM therapy^14^. Notably, a close connection between phospholipase D1 (PLD1) and the Wnt/β-catenin signaling pathway has been reported in CRC and GBM^15–18^.

PLD catalyzes the hydrolysis of phospholipids to generate phosphatidic acid (PA), which activates signaling cascades^19–24^. To date, six mammalian PLD isoforms have been identified and categorized into classical and nonclassical groups, depending on the presence of phox homology (PX) and pleckstrin homology (PH) domains. PLDs are involved in diverse physiological functions, such as intracellular protein trafficking, cytoskeletal dynamics, secretion, membrane remodeling, metabolism, angiogenesis, cell migration, cell proliferation, and stem cell self-renewal^19–24^. PLD1 and PLD2 use phosphatidylcholine (PC) as a substrate and play pivotal roles in various mitogenic and oncogenic signaling pathways, such as G protein-coupled receptors, receptor tyrosine kinases, integrin, mechanistic target of rapamycin (mTOR), and Wnt signaling^25–28^. PLD1 and PLD2 share approximately 50% amino acid homology. In contrast, nonclassical PLDs, including PLD3, PLD4, PLD5, and PLD6, lack the PX and PH domains^21^. PLD1 and PLD2 are transcriptional targets of Wnt/β-catenin and activate its signaling pathway^29–31^. However, unlike PLD2, PLD1 has been well studied for its ability to regulate Wnt/β-catenin and its associated cancer signaling pathways in addition to affecting cancer stemness. Thus, we primarily focused on PLD1 within the context of this review. Hence, this review highlights interactions involving the PLD1 and Wnt/β-catenin signaling cascades in cancer and highlights the possibility of PLD1 as a potential target for combating cancer stemness-mediated chemoresistance.

## Both PLD lipase activity and PLD protein expression modulate cancer-associated signaling pathways

Deregulation of PLD1 is involved in a variety of pathophysiological processes, such as oncogenesis, inflammation, diabetes, thrombosis, multiple sclerosis and neurodegenerative disorders^23,25^. According to The Cancer Genome Atlas, PLD1 is mutated, amplified, and/or upregulated in several types of cancers. *PLD1* was identified as a resident gene in the minimally amplified region of 3q26, which is frequently amplified in several types of cancer and is correlated with a poor prognosis and an invasive phenotype^32^. However, further studies are needed to determine whether targeting these gene-mediated pathways leads to clinical responses and whether 3q26 amplification predicts the response of cancer cells to inhibitors of these pathways.

Physiological activators of PLDs, such as epidermal growth factor (EGF), platelet-derived growth factor, and interleukin-1,β, increase the expression of PLD1 but not PLD2 via enhanced binding of NF-κB to the *PLD1* promoter^33^. Thus, autoregulation of PLD activity might be coupled to selective induction of PLD1 expression via NF-κB and contributes to cancer progression through increased matrix metalloproteinase upregulation and invasion. *CagA*-positive *Helicobacter pylori* infection is a risk factor for the development of severe gastritis and gastric cancer^34^. Infection of gastric cancer cells with cagA-positive *H. pylori* leads to selective induction of PLD1 expression via cagA-dependent activation of NF-κB^35^. PLD1 expression and IκBα phosphorylation are aberrantly increased in *H. pylori*-infected human gastric cancer tissues, and rebamipide, a mucosal-protective antiulcer agent, abolishes *H. pylori cagA*-induced PLD1 expression via inhibition of NF-κB binding to the *PLD1* promoter. Thus, cagA-positive *H. pylori*-induced NF-κB activation might increase the risk of gastric cancer via the upregulation of PLD1 expression. Additionally, mutated *H-Ras* selectively increases the expression of PLD1, but not PLD2, via an interaction between Sp1 and its binding site in the 5′ promoter of *PLD1*^36^. Moreover, overexpression and increased activity of PLD1 are required for mutant H-Ras-induced transformation and tumorigenesis^37^. The Ewing sarcoma (EWS) fusion protein EWS-Fli, which is found in EWS and primitive neuroectodermal tumors, selectively increases PLD2 expression via the ETS binding motif^38^. EWS-Fli1 may play a role in regulating tumor proliferation signaling enzymes via PLD2 expression. Furthermore, both *PLD1* and *PLD2* have been identified as transcriptional target genes of Wnt/β-catenin signaling by binding TCF4/β-catenin to their promoters^27,29,30^. Moreover, PLD isozymes act as positive feedback regulators of Wnt signaling, which subsequently promotes Wnt-driven anchorage-independent growth of CRC cells. Thus, the differential expression of PLD isozymes might be mediated by a distinct signaling pathway or through different transcription factors, depending on the physiological status of the cell or the cell type. Further studies are needed to expand our understanding of the specific mechanisms by which increased PLD expression contributes to cancer development and progression.

Choline generated via the activation of classical PLD is used as a substrate by choline kinase-α (ChK-α), which is upregulated in several cancers and is a major contributor to increased phosphocholine levels, a metabolic hallmark in various cancers^39^. Phosphocholine is the product of the first step in the biosynthesis of PC. Choline is a precursor of the neurotransmitter acetylcholine, the membrane lipids PC and sphingomyelin, and the methyl donor glycine betaine. Choline also acts as an endogenous ligand of the sigma-1 receptor that links both the PLD pathway and cholinergic synapse activity through choline uptake^40^. Choline, an intracellular messenger, links extracellular stimuli to inositol 1,4,5-trisphosphate (IP_3_)-evoked Ca^2+^ signaling through the sigma-1 receptor, which potentiates IP_3_-evoked Ca^2+^ release from the endoplasmic reticulum through IP_3_ receptors^40^. Human gut microbial PLD metabolizes dietary PC and mediates the anaerobic conversion of choline to trimethylamine (TMA)^41^. TMA is further oxidized in the liver to trimethylamine *N*-oxide (TMAO), facilitating its excretion^42^. Elevated plasma TMAO levels have been correlated with various disease states, including colon cancer, cardiovascular disease, diabetes, and nonalcoholic fatty liver disease^41^. Thus, gut bacterial PLD enzymes may represent targets for therapeutic intervention for diseases linked to TMA production. The lack of effect of human PLD inhibitors on bacterial PLD activity suggests the possibility of developing compounds that do not affect the corresponding host PLD enzymes^41^.

PC metabolism affects cell proliferation, migration and signaling, and these mechanisms might be important regulators of tumorigenesis. Targeting the metabolome in cancer is now emerging as a potential therapeutic approach. Thus, ChK-α and PLD1 may be two target enzymes involved in choline phospholipid metabolism in cancer. Combined treatment with ChK-α and PLD inhibitors may be more effective against cancer than individual treatments. Numerous protein targets are present both upstream and downstream of PLD, and most of them respond to mitogenic signals^19,26^. PA generated by PLD activation recruits and activates proteins containing the PA-binding domain. When PLD is activated by EGF, the resulting PA interacts with the PH domain of Sevenless (SOS) and recruits SOS to the plasma membrane, followed by activation of Ras^43,44^. Moreover, PA also binds to Raf-1 and stimulates the Raf-ERK pathway^44^. Additionally, a key target of PA is mTOR, which integrates both nutrient and growth factor signals to control cell growth and proliferation^45,46^. PA binds to the FKBP12-rapamycin binding domain of mTOR and competes with rapamycin-FKBP12^47^. Elevated PLD activity confers resistance to rapamycin in cancer cells^48^. PA regulates various signaling pathways; however, it also alters gene expression. PA suppresses the expression of proapoptotic tumor suppressor genes, including *p53, p21, Egr-1*, and *PTEN*, while PA upregulates the expression of antiapoptotic survival-related genes, such as *Bcl2, cIAP, survivin*, hypoxia-inducible factor-1α (*HIF-1α), MMP-2, MMP-9* and *COX-2*^49–51^. Overall, PLD dysregulation in cancers is related to oncogenic signals and tumorigenesis.

**Fig. 1:**
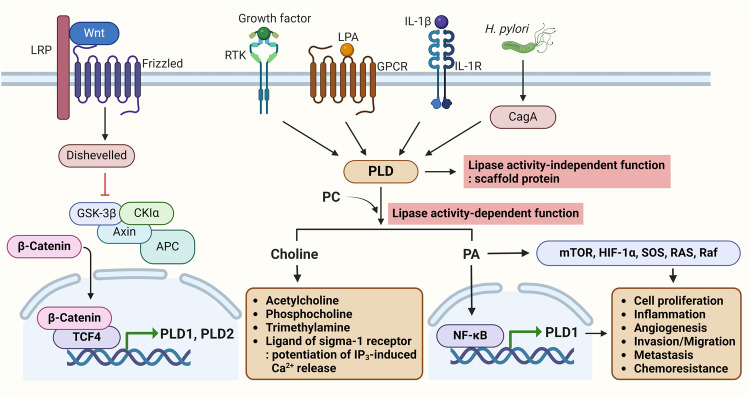
Schematic representation of the roles of PLDs in the modulation of cancer-associated signaling pathways. The activity and expression of PLD are upregulated by various mitogenic and inflammatory pathways, including Wnt/β-catenin, receptor tyrosine kinase (RTK), G-protein coupled receptor (GPCR), IL-1β, and *H. pylori* infection pathways. PLD1 and PLD2 are transcriptional targets of Wnt/β-catenin. PLD modulates cancer-associated signaling pathways via lipase activity-dependent and lipase activity-independent functions. PLD-generated choline is converted to various metabolites and acts as an endogenous ligand of the signal-1 receptor. LRP low-density lipoprotein receptor-related protein, LPA lysophosphatidic acid, IL-1β interleukin-1β.

The interrelationships between PLD and its binding partners enable it to act as a scaffold protein to increase signaling efficiency, integrate and coordinate complex upstream signals, determine which signals are transmitted to downstream pathways, and amplify such downstream signals^52^. The PLD1 protein itself acts as a molecular platform, interacting directly with HIF-1α, prolyl hydroxylase domain-2 protein and von Hippel‒Lindau (VHL) regardless of its lipase activity, thereby dynamically assembling a multiprotein complex that mediates the efficient degradation of HIF-1α in an O_2_-dependent manner^53,54^. HIF-1α is a master transcriptional regulator of the cellular response to hypoxia and increases the transcription of genes involved in angiogenesis, cell survival and proliferation. HIF-1α is a critical mediator of intratumoral heterogeneity, tumor progression, and resistance to therapy in hypoxic tumors. The enzymatic activity of PLD1 is responsible for increased levels of HIF-1α via promotion of protein translation, whereas the PLD1 protein itself destabilizes HIF-1α by interacting directly with the components involved in VHL-dependent degradation of HIF-1α, independent of PLD activity. Overall, PLD1 predominantly promotes HIF-1α translation to induce its upregulation; since the PLD1 protein typically decreases HIF-1α stability, these results suggest that PLD1 plays dual roles in the regulation of HIF-1α. The opposite dual roles of PLD1 in HIF-1α regulation may enable fine-tuning of HIF-1α protein expression. In addition, PLD1 might play a role in accelerating the termination of HIF-1α signaling as soon as PLD activation signals are inactivated, which is an essential mechanism for the strict and rapid regulation of biological responses. Thus, PLD1 might play a crucial role as an oxygen-dependent regulator of HIF-1α stability. Notably, the interaction of the PH domain with these proteins promotes the degradation of HIF-1α independent of the oxygen concentration and suppresses tumor progression, suggesting a novel function of the PLD-PH domain as a therapeutic target against cancer^54,55^. Overall, PLD isozymes modulate cancer-associated pathways and therefore might be utilized in the development of cancer therapeutics (Fig. 1).

## PLD1 coordinates cross-talk between the Wnt/β-catenin and PI3K/Akt pathways

**Fig. 2:**
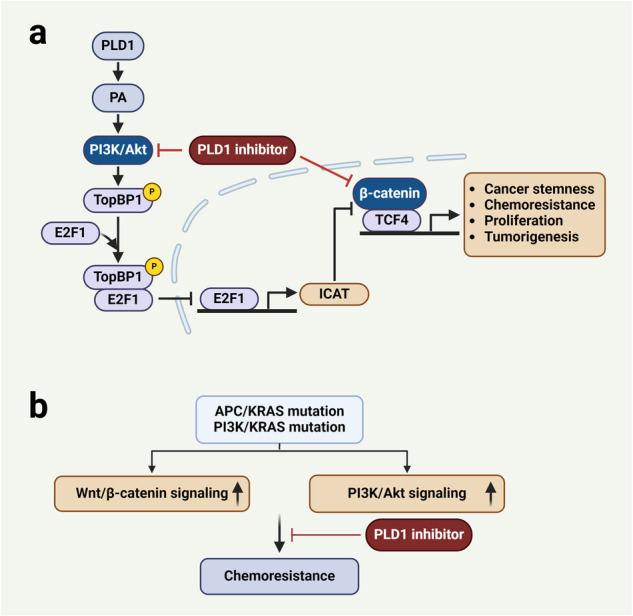
PLD1 mediates cross-talk between the Wnt/β-catenin and PI3K/Akt signaling pathways via the TopBP1-E2F1-ICAT axis. **a** PLD1 coordinates cross-talk between the Wnt/β-catenin and PI3K/Akt signaling pathways. PLD1-generated PA activates PI3K/Akt, which phosphorylates TopBP1. Phosphorylated TopBP1 binds to and suppresses E2F1. Inhibition of E2F1 binding to the *ICAT* promoter suppresses the expression of ICAT. A reduced level of ICAT induces the association of β-catenin with TCF4 and thus activates the Wnt/β-catenin signaling pathway. PLD1 inhibition suppresses Wnt/β-catenin signaling via the upregulation of ICAT. **b** PLD1 inhibition increases the treatment sensitivity of CRC cells with hyperactivation of the Wnt/β-catenin and PI3K/Akt signaling pathways.

The Wnt/β-catenin signaling pathway is one of the aberrantly activated pathways in CRC and GBM and has been reported to regulate the development and maintenance of cancer cells. The destruction complex comprising Axin, adenomatous polyposis coli (APC), glycogen synthase kinase-3 beta (GSK-3β), and casein kinase 1 alpha (CK1α) and disheveled promotes the phosphorylation of β-catenin, leading to its proteasomal degradation^56,57^. In the absence of Wnt ligands, CK1 and GSK-3β sequentially phosphorylate Axin-bound β-catenin. β-TrCP then recognizes and ubiquitylates β-catenin, leading to its rapid degradation by the proteasome. This prevents the activation of β-catenin target genes in the nucleus. Upon the binding of Wnt ligands to the receptor Frizzled and its coreceptors (lipoprotein receptor-related proteins), the Wnt signaling pathway is activated. Wnt signaling inhibits β-catenin ubiquitination, leading to its cytoplasmic accumulation and nuclear translocation. The accumulated β-catenin then translocates into the nucleus, where it binds to and activates T-cell factor (TCF) transcription factors. In the Wnt “off” state, TCFs interact with Groucho transcriptional repressors, preventing gene transcription^58,59^. However, in the “on” state, β-catenin transiently converts TCFs into transcriptional activators of target genes involved in cell proliferation. Most human CRC cases involve somatic mutations in the *APC* tumor suppressor gene, which leads to the activation of Wnt signaling via β-catenin stabilization. Indeed, a variety of cancer-relevant molecules and signaling pathways intersect and may modulate the Wnt pathway. The Wnt/β-catenin and PI3K/Akt signaling pathways are frequently dysregulated in various cancers, including CRC and GBM. The integration of these oncogenic pathways enhances tumor growth and confers resistance to targeted therapies. Accordingly, the identification of critical effectors regulating the multifaceted cross-talk between signaling pathways may lead to the identification of new candidates for the development of targeted therapies useful for CRC management. There is bidirectional cross-talk between the PLD1 and Wnt/β-catenin pathways^27,29,30^. PLD1 inhibition reduces the binding of β-catenin to TCF4 and promotes the association of β-catenin inhibitors and TCF (ICAT) with β-catenin as well as the expression of ICAT, a negative regulator of Wnt signaling. ICAT is known to competitively bind to β-catenin to inhibit β-catenin/TCF-4 complex formation^60^. ICAT is a selective target of PLD1-mediated regulation of the Wnt/β-catenin signaling pathway^60^. Thus, PLD1 may promote Wnt/β-catenin signaling by selectively downregulating ICAT expression. ICAT expression is inversely correlated with the transcriptional activity of β-catenin^61^. ICAT is predominantly expressed in epithelial cells covering villi (where β-catenin is not active in Wnt-induced transcription) and is downregulated in crypt cells (where β-catenin is transcriptionally active). Moreover, PLD1 expression is inversely correlated with ICAT levels in various cancer cells, CRC patient-derived cancer cells, and CRC tissues. Moreover, the *ICAT* gene is downregulated in high-grade glioma tissues compared to low-grade tissues and normal controls, and low ICAT expression indicates a poorer prognosis than high ICAT expression^56^. Targeting PLD1 attenuates spontaneous and colitis-associated intestinal tumorigenesis^16^. Targeting PLD1 upregulated the expression of ICAT in an intestinal tumorigenic mouse model, suggesting the in vivo relevance of the mutual expression of PLD1 and ICAT. ICAT negatively regulates the oncogenic growth of CRC cells. The function of ICAT as an antagonistic regulator of the Wnt/β-catenin pathway suggests that it could be a tumor suppressor gene^62^. PLD-mediated PI3K/Akt activation is responsible for the negative regulation of ICAT expression^16^. E2F1 has been identified as a negative regulator of β-catenin/TCF-dependent transcription^63,64^. However, the underlying mechanism has not yet been identified. Kang et al.^16^ reported that targeting PLD1 enhances the binding of E2F1 to the *ICAT* promoter, which can be abolished by ectopic expression of Akt1. Thus, ICAT (a transcriptional target of E2F1) may act as a crucial node in the link between E2F1 and β-catenin signaling, and the PLD1-PI3K/Akt pathway negatively regulates ICAT expression via the suppression of E2F1 binding to the *ICAT* promoter. It has been reported that the PI3K/Akt signaling pathway regulates E2F1 through the E2F1-interacting protein TopBP1 (topoisomerase IIβ−binding protein), which inhibits E2F1-dependent apoptosis^65^. Phosphorylation of TopBP1 by Akt is crucial for the interaction of TopBP1 with and repression of E2F1^65^. Inhibition of PLD1 and PI3K abolishes the phosphorylation of TopBP1 and the interaction of TopBP1 with E2F1, suggesting the involvement of PI3K/Akt1 and TopBP1 in the regulation of PLD1-mediated ICAT expression^16^. Analysis of the signaling events involved in PLD1-mediated β-catenin/TCF-4 activity revealed a previously unknown link between the β-catenin/TCF-4 pathway and the PI3K/Akt-TopBP1-E2F1-ICAT pathway (Fig. 2a). Notably, PLD1 inhibition may promote ICAT expression by downregulating retinoblastoma tumor suppressor (RB1) expression and inducing the expression of the free form of E2F1, which is regulated by the PI3K/Akt-TopBP1 pathway. Moreover, the PI3K/Akt-TopBP1-E2F1 pathway is involved in PLD1-mediated transactivation of β-catenin/TCF4, and E2F1 is required for PLD1 inhibition-induced suppression of TCF transactivation. E2F1-induced ICAT expression is functionally required for E2F1-mediated inhibition of Wnt/β-catenin oncogenic signaling. Thus, the PI3K/Akt-TopBP1-E2F1-ICAT signaling axis is involved in PLD1-mediated regulation of β-catenin/TCF transactivation. PLD1, which is linked to ICAT, mediates molecular cross-talk between the Wnt/β-catenin and PI3K/Akt pathways and thus could be proposed as a novel prognostic biomarker for CRC. *APC* mutations are risk factors for patients treated with PI3K/Akt pathway inhibitors^66^. It has been suggested that β-catenin confers resistance to PI3K and Akt inhibitors in CRC^67^. Thus, oncogenic activation of the Wnt/β-catenin pathway could be a mechanism of resistance to PI3K and Akt inhibitors. Patient-derived xenograft (PDX) models reveal certain pathological and molecular features of the original disease^68^. Treatment with PLD1 inhibitors significantly decreases tumor growth in mice bearing PDXs with *PIK3CA* and *KRAS* mutations and in mice bearing PDXs with *APC* and *KRAS* mutations^16^. Moreover, compared with vehicle treatment, PLD1 inhibition increased ICAT expression and reduced the expression of Wnt target genes in tumor tissues from the PDX group. The hyperactivation of the Wnt/β-catenin and PI3K/Akt signaling pathways has limited clinical benefit mostly due to unknown resistance mechanisms and the lack of predictive biomarkers of drug response. Thus, PLD1 inhibition linked to the upregulation of ICAT might increase the treatment sensitivity of CRC cells with hyperactivation of the Wnt/β-catenin and PI3K/Akt signaling pathways (Fig. 2b). The clinical development of PLD1 inhibitors may assist in the treatment of patients with CRC carrying *APC* and *PI3KCA* mutations.

## Targeting PLD1 reduces the self-renewal capacity of CSCs and suppresses chemoresistance via the miR-192/4465–RB1-E2F1 and E2F1–miR-4496-β-catenin signaling axes

**Fig. 3:**
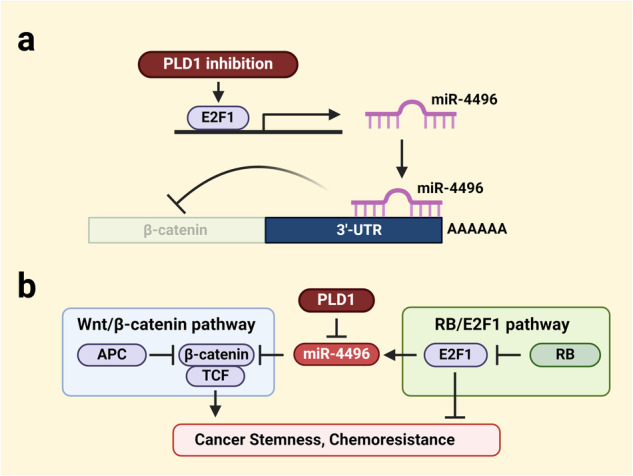
miR-4496, a new linker between the Wnt/β-catenin and RB1/E2F1 pathways, suppresses cancer stemness and chemoresistance. **a** PLD1 promotes the binding of E2F1 to the promoter of miR-4496 and induces the expression of miR-4496, which downregulates β-catenin at the posttranscriptional level. **b** miR-4496 acts as a molecular linker connecting the RB1-E2F1 and Wnt/β-catenin signaling pathways and suppresses cancer stemness and chemoresistance.

**Fig. 4:**
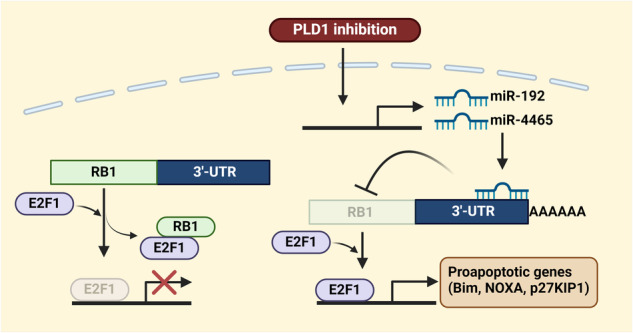
PLD1 inhibition promotes E2F1-induced apoptosis via retinoblastoma tumor suppressor (RB1) downregulation. PLD1 inhibition increases the expression of miR-192/−4465 and downregulates that of RB1. Downregulation of RB1 causes the release of E2F1 from the RB1-E2F1 complex, thereby allowing the transcriptional activation of E2F1. Ultimately, E2F1 induces apoptosis via the expression of proapoptotic E2F1 target genes.

**Fig. 5:**
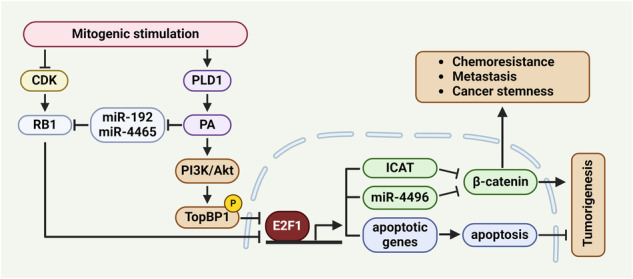
PLD1 as a multitarget regulator of cancer-related pathways. Mitogenic stimulation upregulates the expression and activity of PLD1. PA activates PI3K/Akt and phosphorylates TopBP1, which binds to and suppresses E2F1 expression. PA downregulates miR-192/−4465 expression by targeting retinoblastoma tumor suppressor (RB1). A PLD1 inhibitor induces the release of E2F1 via the downregulation of RB1 and the TopBP1-E2F1 complex. The free form of E2F1 promotes apoptosis by inducing the expression of proapoptotic E2F1 target genes. Moreover, the transcriptional activation of E2F1 induced by a PLD1 inhibitor downregulates β-catenin by increasing miR-4496 and ICAT levels. Ultimately, downregulation of β-catenin by PLD1 inhibitors suppresses cancer stemness, drug resistance, and tumorigenesis.

Targeting PLD1 in *Apc*^*Min/+*^ and colitis-associated CRC mice decreases the level of nuclear β-catenin but not membrane-associated β-catenin, which is detected both in the nontransformed areas of the intestines and in the normal areas of *Apc*^*Min/+*^ mice^15^. Intestinal epithelial cell-specific PLD1 overexpression in *Apc*^*Min/+*^ mice increases nuclear β-catenin levels and tumor development. β-Catenin is mainly regulated at the protein level. Depletion and inhibition of PLD1 decrease β-catenin mRNA levels and suppress the expression of its target genes^15^. Moreover, high mRNA expression of β-catenin is correlated with a poorer prognosis in CRC patients. Targeting PLD1 downregulates β-catenin expression via E2F1-induced miR-4496 upregulation, suggesting dynamic changes in β-catenin levels by PLD1 and a new pathway by which E2F1 represses β-catenin-TCF activity. Targeting PLD1 increases the promoter activity of miR-4496 and promotes the binding of E2F1 to its promoters^15^. Thus, miR-4496 is considered a new target of E2F1 that represents a crucial node responsible for mediating the crosstalk between E2F1 and Wnt/β-catenin signaling. Moreover, miR-4496 is a new miR that targets β-catenin (Fig. 3a). Thus, miR-4496 acts as a new linker molecule mediating cross-talk between the Wnt/β-catenin and RB1/E2F1 pathways in CRC (Fig. 3b). Targeting PLD1 greatly decreases the proportion of CD44^high^CD133^high^ cells, decreases sphere-forming capacity, and suppresses proliferation, and all of these effects can be attenuated by E2F1 depletion and anti-miR-4496^15^. E2F1 is a key downstream target of the RB1 protein. The interaction of RB1 with E2F1 decreases E2F1 transcriptional activity and inhibits hyperphosphorylation of RB1 by cyclin-dependent kinases, causing the escape of RB1 from E2F inhibition, thereby allowing transcriptional activation. E2F1 can function as both a tumor suppressor and an oncogene under different conditions^69^. E2F1 may function as a tumor suppressor in CRC^64^. E2F activation is the ultimate consequence of RB pathway deregulation. *RB1* in CRC is not mutated but may be amplified^70,71^. In CRC, RB1 is more likely to act as an oncoprotein than a tumor suppressor. Notably, the downregulation of RB1 by targeting PLD1 releases active E2F1 and induces E2F1 transactivation. PLD1 provides a previously unknown linker between the Wnt/β-catenin and E2F1 signaling pathways via miR-4496. Ultimately, PLD1 governs the self-renewal capacity of cancer-initiating cells (C-ICs) through the E2F1–miR-4496–β-catenin axis (Fig. 3b). PLD1 inactivation decreases the expression of C-IC markers and the self-renewal capacity of C-ICs for serial tumor initiation and suppresses chemoresistance^15^. Chemoresistance mediated by C-ICs has emerged as an important cellular property that enables tumors to reoccur after initial cytoreductive therapy. Compared with PLD1 depletion or drug treatment, treatment with anticancer drugs, such as 5-fluorouracil and oxaliplatin, in PLD1-depleted cells increases the chemosensitivity and self-renewal capacity of colon C-ICs. However, the key modulators and mechanisms underlying the functional interactions between signaling circuits in cancer remain elusive. CSCs, which are associated with tumor relapse and progression, are considered responsible for the poor outcomes of various cancers. Targeting PLD1 increases the expression of apoptotic genes but decreases the level of RB1 via miR-192 and −4465^18^. The apoptosis induced by PLD1 inhibition is prevented by RB1 overexpression, suggesting that PLD1 inactivation-induced apoptosis is closely associated with the downregulation of RB1. Anti-miR-192/4465 treatment suppressed PLD1 inactivation-induced expression of pro-apoptotic E2F1 target genes and promoted apoptosis (Fig. 4). PLD1 expression is inversely correlated with the levels of E2F1 target genes in the survival of CRC patients; thus, PLD1 and E2F1 might be potential prognostic biomarkers and therapeutic targets in CRC. The expression of RB1, Akt1, and miR-192/4465 reduces PLD1 depletion-induced chemosensitivity^18^. PLD1 targeting-induced β-catenin downregulation is reversed by ectopic expression of RB1, Akt1, and anti-miR-192/4465. PLD1-mediated cancer-initiating capacity is associated with the upregulation of β-catenin and its target genes. PLD1 inhibition promotes E2F1-dependent apoptosis through the miR-192/4465–RB1-E2F1 and Akt/TopBP1-E2F1 signaling pathways. Thus, PLD1 regulates the apoptosis and self-renewal capacity of C-ICs via the modulation of cross-talk involving the miR-192/4465–RB1–E2F1, PI3K/Akt–TopBP1-E2F1, and E2F1–miR-4496-β-catenin signaling pathways (Fig. 5). Targeting these signaling networks is particularly attractive because almost all human cancers exhibit alterations involving these pathways. Considering the highly complex interactions among cancer-relevant pathways, targeting these pathways via PLD1 inhibition may be an effective therapy for CRC patients.

## PLD1 as a potential target of resistance to temozolomide in GBM

**Fig. 6:**
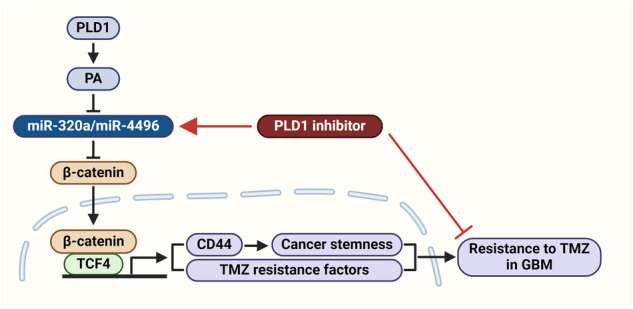
A PLD1 inhibitor increases the treatment sensitivity of GBM cells with temozolomide (TMZ) resistance via downregulation of β-catenin. PLD1 stabilizes the level of β-catenin and increases the binding of β-catenin to TCF4, followed by upregulation of CD44 and TMZ resistance-related factors, which contribute to resistance to TMA in GBM. PLD1 inhibitors downregulate β-catenin via upregulation of miR-320a and miR-4496 and thus overcome resistance to TMZ by downregulating CD44 and TMZ resistance-related factors.

GBM is a highly aggressive brain tumor comprising tumor-propagating GSCs that promote therapeutic resistance and tumor recurrence and has a poor prognosis. Thus, the identification and control of new target proteins responsible for drug resistance is an effective approach for overcoming therapeutic resistance. PLD has been reported to be closely associated with signaling pathways modified in GBM^72–75^. The PI3K/Akt and Wnt/β‐catenin signaling pathways are associated with glioma development and a poor prognosis in GBM patients^76^. The alkylating drug temozolomide (TMZ) is used clinically to treat GBM patients. Patients with recurrent GBM who develop TMZ resistance have limited therapeutic options. Elucidating the molecular basis of TMZ resistance may contribute to the development of logically designed combination therapies that block resistance to TMZ chemotherapy. Recurrent GBM is characterized by resistance to radio/chemotherapy and a poor clinical prognosis. PLD1 expression is significantly greater in recurrent GBM tumors than in primary GBM malignancies. TMZ significantly upregulated the expression of PLD1 in CD44^Low^ and CD44^Moderate^ GSCs but not in CD44^High^ cells, in which PLD1 is highly expressed. PLD1 confers resistance to TMZ in CD44^High^ GSCs and increases their capacity for self-renewal and maintenance. PLD1 may play a tumorigenic role by enriching the GSC pool. Therefore, PLD1 may serve as a novel target for the treatment of GSCs. PLD1 suppression significantly influences the outcome of radiotherapy. PLD1 inhibition specifically targets GSCs but not neuroprogenitor cells. PLD1 depletion sensitizes GSCs to the effect of TMZ on GSC‐derived intracranial tumors^17^. PLD1 expression is correlated with TMZ resistance-related factors (*MGMT, ABCB1, ABCG2, PHF6, MMP16*, and *MCL1*) in GBM tumors. However, the molecular mechanisms underlying TMZ resistance in GBM remain unclear. The upregulation of miR‐320a and miR‐4496 by PLD1 inhibition attenuates self‐renewal, tumor‐initiating capacity, and chemoresistance^15,17^. miR‐320a and miR‐4496 have target site(s) in the 3′‐untranslated region of TMZ resistance genes. PLD1 inhibition, but not TMZ treatment, reduces the levels of TMZ resistance-related factors via upregulation of these miRNAs and sensitizes GSCs and GSC-derived intracranial tumors to TMZ. miR‐320a and miR‐4496 induced by PLD1 depletion downregulate the expression of *MCL1*, an antiapoptotic gene that is a key mediator of cell survival and drug resistance in GBM^77^. The Wnt/β‐catenin signaling pathway reportedly promotes stem cell properties and resistance to radio/chemotherapy in GBM^78,79^. miR-320a and miR-4496 induced by PLD1 inhibition downregulate β‐catenin and reduce the self-renewal capacity of CSCs in CRC and gastric cancer^15,80,81^. Thus, targeting PLD1 may suppress chemoresistance via downregulation of the Wnt/β‐catenin signaling pathway in various cancers, including GBM, CRC and gastric cancer. Moreover, PLD1 inhibition sensitizes GSC‐derived intracranial tumors to TMZ via miR-4496, supporting the clinical application of this combination therapy for the treatment of GBM^17^. The PLD1–miR‐4496 axis acts as a new target of TMZ resistance, and targeting this axis might represent a potential therapeutic strategy against GSC‐derived GBM tumorigenesis (Fig. 6). Tumor recurrence has also been linked to epigenetic mechanisms and cellular pathways^82^. Epigenetic modulators such as histone deacetylase (HDAC) inhibitors are promising anticancer agents. Recently, it was reported that PLD1 acts as a transcriptional target of HDAC inhibitors such as vorinostat and confers resistance to vorinostat in GBM^73^. Vorinostat-induced PLD1 upregulation plays a pivotal role in protection against apoptosis in GBM. The combination of drugs significantly suppresses cell invasion, angiogenesis, colony formation, and self-renewal capacity and intracranial tumor formation in the context of GBM. As PLD1 is a novel target of vorinostat resistance, combination therapy with PLD1 inhibitors and vorinostat may represent a potential therapeutic strategy against GBM tumorigenesis. PLD1‐based TMZ resistance mechanisms in GSCs represent crucial nodes for therapeutic intervention. Thus, PLD1 inhibition may overcome TMZ resistance and represents a potential treatment strategy for GBM patients.

## Challenges of PLD signaling-targeted drugs in cancer

**Fig. 7:**
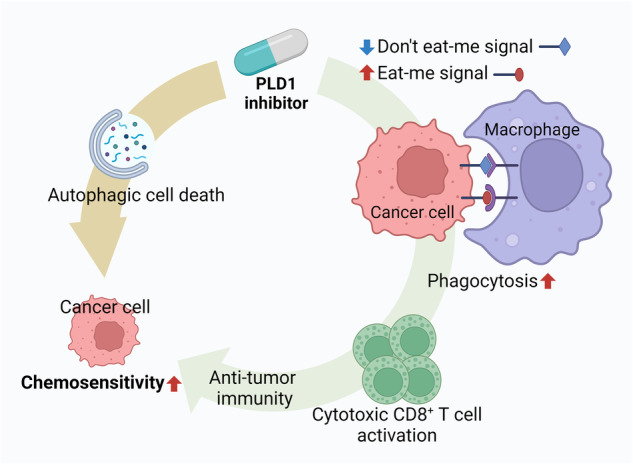
A hypothetical model for the efficacy of PLD1 inhibitors in increasing chemosensitivity. PLD inhibitors induce immunogenic cell death and autophagic cell death and ultimately increase chemosensitivity. PLD1 inhibitors promote macrophage-mediated phagocytosis via modulation of “eat-me” and “don’t-eat-me” signals and subsequently induce antitumor immunity via cytotoxic CD8^+^ T-cell activation.

The incorporation of molecular targeted agents has substantially increased the success rate of treating cancer patients with positive expression of target genes. However, as with conventional chemotherapeutics, resistance is also an obstacle for molecular-targeted agents. In addition to the common mechanisms of multidrug resistance, redundancy in signaling pathways in cancer cells is an important mechanism of resistance to molecularly targeted agents. The substantial effect of PLD on the hallmarks of cancer, stemness, and chemoresistance makes it an attractive target for cancer therapy. Efforts to develop small molecules as specific PLD inhibitors have been made over the past 15 years. The identification of halopemide as a PLD inhibitor by a group at Novartis was an important contribution to the PLD field^83^. Halopemide, originally reported by Janssen, is an atypical antipsychotic drug that achieves high plasma exposure without causing adverse events^84^. Thus, it is suggested that the inhibition of PLD1 and PLD2 by halopemide and its analogs is safe in humans. This represents a major step toward decreasing the risks associated with therapeutic approaches. PLD isozymes were once thought to be undruggable targets, owing to the broad involvement of PA in cell signaling and concerns that such inhibitors would be too toxic for use in humans. Using halopemide as a starting molecule, Scott et al. developed a series of isozyme-selective PLD inhibitors through diversity-oriented synthesis and an iterative analog library synthesis approach for lead optimization^85^. VU0359595 and VU0364739 were developed as the first highly PLD1-selective and PLD2-selective inhibitors, respectively^85,86^. Isozyme-selective PLD inhibitors have better ancillary pharmacological profiles than halopemide and exhibit attractive drug metabolism and pharmacokinetic profiles suitable for in vivo proof-of-concept studies^84,86,87^. PLD2 has also emerged as a target for cancer and other diseases, and VU0364739 has served as a proof-of-concept compound^74,88,89^. Since other PLD isoforms, especially PLD2, may act as redundant mediators of PLD1^90^, the development of resistance to PLD1 inhibitors *via* redundancy has emerged as a challenge. The development of dual PLD1 and PLD2 inhibitors may be a strategy to overcome such redundancy in PLD signaling. If PLD2 confers redundancy by generating PA in the absence of PLD1, PLD2-derived PA may mediate the regulation of cancer signaling by PLD1. In addition to the enzymatic activity of PLD, PLD1- or PLD2-specific binding proteins and the modulation of other regulatory factors may affect distinct cancer signaling pathways mediated by PLD isozymes. Long-term PLD activity is involved in the upregulation of PLD1 expression but not PLD2 expression^33^. Thus, long-term treatment with PLD inhibitors might negatively modulate the interaction between PLD1 and other signaling factors. It is suggested that the major mechanism of the anticancer effect of the PLD1 inhibitor A3373 is the inhibition of PA production, which may affect the interaction between PLD1 and its binding factors. Although PLD1 has been well studied for its ability to regulate cross-talk between the PI3K/Akt and Wnt/β-catenin pathways, future studies are needed to further investigate the involvement of PLD2 in these signaling pathways. Moreover, a PLD1 inhibitor was found to sensitize cancer cells to autophagy inhibition^91^. PLD coordinates with major players in the autophagic pathway. The role of autophagy in cancer and its responsiveness to treatment is complicated. PLD1 inhibition augments the efficacy of anticancer regimens by facilitating autophagic pathways^91^. Controlling autophagy may also be used as a therapeutic strategy to treat cancer cells that are resistant to cell death (Fig. 7). Blockade of autophagy can be detrimental to the survival of cancer cells in which autophagy is activated; PLD was recently found to be related to the molecular machinery regulating autophagy, so inhibiting autophagy could represent a new therapeutic approach to enhance the anticancer efficacy of PLD inhibition. Consequently, recent studies on PLD inhibitors have revealed the critical role of PA in cancer pathophysiology, suggesting an unconventional approach to cancer treatment. Recently, two research groups reported the structures of human PLD1 (hPLD1) and hPLD2 without the N-terminal PX-PH domains^92,93^, which are critical for the binding of PLD to proteins and lipids. Structure-based design revealed the basis of isozyme selectivity and demonstrated how these structures can accelerate drug discovery efforts to improve PLD inhibitor design. This work not only informs further investigations of PLD enzymes as therapeutic targets but will also be necessary for the development of the next generation of PLD inhibitors. Recently, we developed a selective PLD1 inhibitor, A3373, using computer-aided drug design and pharmacophore studies^31^. The PLD1 inhibitor promoted the apoptosis of CRC cells but not that of normal colonic mucosa cells, and it exhibited no cardiotoxicity. Oral administration of A3373 was effective. A3373 had a long half-life (3.2 and 3.8 h after intravenous and oral dosing, respectively), high plasma concentration (area under the curve values of 6,316.8 and 13,647.5 ng/h/mL after intravenous and oral dosing, respectively) and high oral bioavailability (108.0%) in an in vivo pharmacokinetic study^31^. Immunotherapy is a new frontier in cancer treatment. Among numerous immunogenic strategies, the induction of immunogenic cell death (ICD) in cancer cells has emerged as one of the most effective strategies to overcome cancer^94^. Induction of ICD represents a new strategy for overcoming tumor immune evasion. During ICD, apoptotic cancer cells are taken up by macrophages and processed into tumor-associated antigens, activating adaptive immunity^95^. Aberrant activation of Wnt/β-catenin signaling is linked to an immunosuppressive tumor microenvironment and resistance to immune checkpoint blockade in different cancer subtypes^96^. The PLD1 inhibitor attenuated colitis-associated CRC and orthotopically injected tumors, probably by controlling multiple pathways, including Wnt signaling, phagocytosis checkpoint, and immune signaling pathways. A3373-treated CRC cells induced ICD, which was accompanied by downregulation of “don’t-eat-me” signals and upregulation of “eat-me” signals, subsequently promoted the phagocytosis of cancer cells by macrophages. Ultimately, PLD1 inhibitor-treated cancer cells were more susceptible to cytotoxic T-cell-mediated killing (Fig. 7). Inhibition of PLD1 by A3373 decreased the self-renewal capacity of CRC cells by downregulating the Wnt/β-catenin signaling pathway, and PLD1 inhibitor-induced apoptosis led to increased activation of macrophages and cytotoxic CD8^+^ T cells^31^. Immune checkpoints, such as programmed cell death ligand 1 (PD-L1), are related to resistance to antitumor therapies and are involved in immune escape^97^. Compared with monotherapy, combination therapy with A3373 and αPD-L1 further enhances antitumor immunity in the tumor microenvironment. Thus, PLD1 inhibition may overcome the limited clinical benefits associated with drug resistance. Thus, PLD1 inhibitors may function as potential immunotherapeutic agents based on ICD and immune activation and may offer a promising new treatment modality for cancer immunotherapy.

## Conclusions and future perspectives

In this review, we highlighted the key features and mechanisms of PLD1 regulation in CSC maintenance and subsequent drug resistance. Mutations and hyperactivation of the Wnt/β-catenin signaling pathway in several cancer cell types and CSCs often induce drug resistance and recurrence in patients with malignancies. Notably, close cross-talk occurs between the Wnt/β-catenin signaling pathway and several other signaling pathways. PLD1 mediates cross-talk between the Wnt/β-catenin and PI3K/Akt pathways and activates Wnt/β-catenin signaling pathways via the PI3K/Akt-TopBP1-E2F1-ICAT axis. miR-4496 acts as a molecular linker between the Wnt/β-catenin and RB1-E2F1 pathways. PLD1 inhibition suppresses cancer stemness and chemoresistance via miR-192/4465–RB1-E2F1 and E2F1–miR-4496–β-catenin. PLD activity is now strongly linked to various cancers and other diseases, such as neurodegenerative and inflammatory diseases. As our understanding of the potential of PLD for druggability grows, the hope that PLD might be a drug target is becoming closer to reality. A newly developed PLD1 inhibitor, A3373, based on its structure, may act as a promising immune modulator in cancer treatment via ICD induction. This study provides new insights into the function of PLD1 in the tumor microenvironment and provides a good rationale for treating recurrent tumors.
